# International biobanks: do or do not?

**DOI:** 10.4155/fso.15.75

**Published:** 2015-09-22

**Authors:** Paul N Span, Peggy Manders

**Affiliations:** 1 Department of Radiation Oncology, Radboud University Medical Center, Nijmegen, The Netherlands; 2 Radboud Biobank, Radboud University Medical Center, Nijmegen, The Netherlands

**Keywords:** biobank, clinical data, disease, ethics, multicenter, tissue

There is no question that biobanks have become essential in clinical research. Clinicians and researchers alike recognize that patient-tailored precision medicine can only be performed after large-scale investigation of well-powered datasets, in which data from complex, multifactorial diseases have been meticulously defined [1]. In order to collect biomaterial and data from sufficient subjects and/or patients, many biobanks are now multi-institutional or even multinational. This comes with specific issues, mainly concerning definitions and standardization, but also diversity in ethics and regulations.

## Biobank

First off, what constitutes a biobank? The most conventional definition is a collection of biomaterial plus associated clinical data [2]. The biomaterial can be any material collected from a subject, but most often consists of blood and/or DNA, and sometimes tissue from a diseased site such as a tumor. For the sake of this editorial we only discuss human biobanks, but biobanks can also be derived from experimental animal studies, or even cell lines. Biobanks can be population-based or may be disease-specific [3]. In short, population biobanks are large, organized, searchable repositories that are national or international in scope. Over a number of years, these collections aim to follow a cohort of individuals to assess the impact of a variety of factors (e.g., income, lifestyle and environment) on disease status and their progression. Disease-specific biobanks have the advantage of offering insight into the genetic basis of rare conditions as well as that of more common diseases [3]. For most of this editorial we will focus on disease-specific biobanks.

## Biomaterial

The biomaterial and its procurement, preservation and analysis have to be strictly defined and standardized, especially in multicenter and international biobanks. Not only does this standardization have to be defined *a priori*, but also a quality control system has to be in place to monitor the adherence to definitions and performance of the participating centers. Only by proper standardization does the biobank contain sufficient and accurate data for further investigations. Whatever biomaterial is collected, it has to be related to the scientific purposes of the biobank. A problem with this might come from the fact that often biobanks collect samples over many years and thus the material itself plus the standard operating procedures have to be defined in a time when the eventual use is not yet clear. In our own experience, the collection of tumor RNA from a large number of breast cancer patients over the years using a standard column-based isolation kit lead to a biobank that was not useful for the investigation of miRNAs, which could not have been envisioned at the start of the biobank. Techniques (e.g., nucleotide isolation kits and sequencing equipment) will improve over time. Therefore, predefined standard operating procedures might be outdated, and the biobank will have to decide whether to upgrade and take advantage of new possibilities, or to adhere to the earlier protocols for the sake of standardization.

Availability of equipment and resources can also lead to unavoidable differences in protocols and quality of the biomaterials between participating institutions and/or countries. Maintenance of consistent intersite processing can be challenging. In this case, centralization of the processing and/or storage of the collected biomaterial might be an option to consider. However, in studies where samples are shipped to a different location for processing, the time delay between collection and stabilization may lead to loss of some unstable markers [4]. Thus, depending on which type of variation is the most crucial for the biobank, a central or a decentralized system (or a hybrid of these) should be attained.

## Clinical data

Similarly, clinical data are in fact relevant personal and health information (which may include health records, family history, lifestyle and genetic information) from a patient that is relevant for the scientific purpose of the clinical biobank. A minimal dataset will have to be defined containing all possibly relevant information. On the other hand, the items should be manageable and obtainable from most, if not all, contributing institutions. It is the hallmark of precision medicine for which this data are necessary. Only by collecting all relevant data and correlating this with outcome will future patient-tailored treatment be feasible.

Concerning intricacies in the collection of clinical data especially relevant in international biobanks, special attention should be given to nomenclature and definitions: are all participants talking about the same thing? Strictly define your parameters. Collect as much raw data as possible; so, register dates of diagnosis, surgery, start and end of treatment, recurrence and/or death, rather then asking the participants to report, for example, recurrence-free survival. Additionally, be aware of differences in standard treatment, follow-up features and interval, etc. A document should be in place defining all parameters and criteria, relevant for the biobank and its scientific purpose and as agreed upon by all participants [5].

An important matter of concern will be the vastly increasing amount of data, especially sequencing data. Information and communication technology solutions for data storage, with appropriate backup systems, should be accessible by all using a variety of systems, including in the future. For retrospective biobanks this can have the consequence that old paper-based patient files would have to be transferred to electronic systems. Even relatively current patient file systems might be incompatible with the scientific biobank data storage system, making the transfer of data challenging. This can be a costly aspect of a biobank, for which resources should be allocated beforehand.

## Cost

Indeed, it is not only expensive to set up a biobank, but also to operate and maintain. For international biobanks, the cost of particular commercial kits (such as for DNA isolation, etc.) might be prohibitive for some institutions in low-income countries with poor research funding. However, even for well-funded organizations, funding for biobanks is often for a predefined period of time and purpose. Therefore, it is important for biobanks to adopt a business model in which both cost and revenue is considered. However, commercialization of the biobank biomaterial and/or clinical data is not always feasible, either due to national or institutional regulations, due to ethical guidelines and/or because subjects refuse in an informed consent.

## Ethics

For all types of biobanks, an informed consent should be in place, and attention should be given to the patient's right to withdraw, what to do with unsolicited findings, how to deal with privacy issues and whether commercialization is permitted, etc. [3]. A particular problem with decentralized international biobanks is the fact that ethical guidelines and the patient's attitude toward biobanks can differ widely between countries [6]. Initially, all ethical considerations should be in line with national guidelines in the country where the patient resides and the biomaterial is obtained. However, there is no global consensus regarding sharing biomaterial and data. Eventually, consent will become ongoing and dynamic; participants will be able to engage as much as they choose and alter their consent choices over time [3].

## Patient participation

It is important to realize that a patient or subject can play different roles in a biobank: not only as a donor, but also active in setting up the biobank; and helping devise the informed consent, the scientific purpose and the publicity surrounding the biobank. A patient can have different questions concerning his disease compared with researchers. Also, they need to know biobanks exist and why they exist [6]. Most objections against biobanks are born out of mistrust in the objectives of such a biobank initiative, which might be thwarted by a strong and early patient participation in the above-mentioned aspects of the biobank.

## Conclusion

Biobanks are crucial in many aspects of medical practice and research. However larger, multi-institutional or multinational initiatives are difficult to devise correctly. Start a biobank, but do it right: ‘There is no try.’
